# Neural correlates of semantic and syntactic processes in the comprehension of case marked pronouns: Evidence from German and Dutch

**DOI:** 10.1186/1471-2202-7-23

**Published:** 2006-03-09

**Authors:** Monique JA Lamers, Bernadette M Jansma, Anke Hammer, Thomas F Münte

**Affiliations:** 1 Department of Linguistics, Radboud University Nijmegen, The Netherlands; 2 Faculty of Psychology, Department of Neurocognition, Maastricht University, Maastricht, The Netherlands; 3 Department of Neuropsychology, Otto-von-Guericke-Universität Magdeburg, Germany

## Abstract

**Background:**

It is well known that both semantic and syntactic information play a role in pronoun resolution in sentences. However, it is unclear what the relative contribution of these sources of information is for the establishment of a coreferential relationship between the pronoun and the antecedent in combination with a local structural case constraint on the pronoun (i.e. case assignment of a pronoun under preposition governing). In a prepositional phrase in German and Dutch, it is the preposition that assigns case to the pronoun. Furthermore, in these languages different overtly case-marked pronouns are used to refer to male and female persons. Thus, one can manipulate biological/syntactic gender features separately from case marking features.

The major aim of this study was to determine what the influence of gender information in combination with a local structural case constraint is on the processing of a personal pronoun in a sentence.

Event-related brain potential (ERP) experiments were performed in German and in Dutch. In a word by word sentence reading study in German and Dutch, gender congruency between the antecedent and the pronoun was manipulated and/or case assignment by the preposition was violated while ERPs of young native speakers were recorded.

**Results:**

The German and the Dutch ERP data showed an enlarged negativity broadly distributed starting approximately 350 ms after onset of the pronoun followed by a late positivity for gender violations. For syntactic incongruencies without gender violations only a positivity was present. The Dutch data showed an earlier onset of the positivity in comparison to German.

**Conclusion:**

Finding negativities and positivities for conditions with a gender violation indicates that pronoun resolution with gender incongruency between the pronoun and the antecedent suffers from semantic as well as syntactic integration problems. The presence of a positivity for the syntactically incongruent conditions without gender violations suggests that the processing of incorrect case marking without a gender violation gives rise to syntactic but not semantic integration problems. We suggest that the more prominent case violation in Dutch caused the earlier onset of the positivity in the Dutch study. In addition, the pattern of ERP effects shows that both case and gender information are used almost immediately implying that the local structural constraint affects the resolution process with more processing activity than for a pronoun of which only one source of information is violated or incongruent.

## Background

An essential part of discourse comprehension is building cohesive links between sentences and sentence fragments. Pronouns are devices with which these cohesive links can be established. Because pronouns normally refer back to an element earlier mentioned in the sentence representing the same entity, a coreferential relationship has to be established between the pronoun and the antecedent. This is not a trivial process and consists of determination, (re)activation and interpretation of the antecedent as well as integrating the pronoun itself into the sentence structure built up so far [1-4]. The studies presented in this paper address both processes. More specifically, the influence of gender congruency between the pronoun and the antecedent on the establishment of the coreferential relationship will be investigated, as well as the influence of a local structural case constraint on the pronoun (i.e. case assignment of a pronoun under preposition governing), using event related brain potentials.

Psycholinguistic research of the last two decades revealed that on-line pronoun resolution may draw on diverse sources including discourse and pragmatic information, syntactic processes and syntactic constraints as well as lexical/semantic phenomena (cf. ([3,5-7]). These claims are based on different methodologies varying from questionnaires, self-paced reading studies (with and without grammatical judgement tasks) to cross-modal priming and event-related brain potential measurements. This might be one reason for the diverging results. There is, however, some consensus that the resolution process is facilitated if certain constraints are met [8,9,4]. It seems obvious that gender information plays a major role in the resolution process of personal pronouns referring to male or female persons (i.e. the man, the woman). According to generative grammar rules pronouns have the same index as the antecedent [10,11], thus inheriting the gender (masculine, feminine, or neutral), and number (singular and plural) characteristics of the antecedent. Hence, the pronoun and the antecedent agree in number and gender. This helps to determine the antecedent and facilitates the comprehension process [5]. Although agreement constraints are induced by grammatical rules, they clearly reflect certain semantic/conceptual characteristics as well (cf [12-14]).

Recently, event related brain potentials (ERPs) have been used to investigate pronoun resolution. ERPs provide information about the time-course of brain activity related to pronoun processing without introducing an extra unrelated task [15]. ERPs are multidimensional in nature. Because of the high temporal resolution they have been proven to be extremely useful determine the time course of different ongoing processes in language comprehension. In contrast to overt responses that are usually obtained with some delay and possible contamination by decision related processes, ERP effects ("components") are an immediate expression of the functional changes of the brain which can be measured in parallel with the comprehension processes. Effects are described that primarily index the processing of semantic/conceptual information and syntactic information. An enhanced negative effect with a peak around 400 ms post-stimulus largest over central and right posterior electrode sites is found for difficulties related to semantic integration processes ("N400" [16-19]) as well as for discourse integration [20,21]. In relation to syntactic processing a late positivity with an onset of approximately 500 ms with a maximum at 600 ms after onset of the critical word at centroparietal sites has been reported ("P600"). The problems that elicit this late positivity vary from syntactic violations such as agreement violations [22], phrase structure violations [23,24] as well as reanalysis of a garden-path sentences or (sub)processes involved in computing a less-preferred structure in local structure ambiguity resolution [25-27] and structure complexity [28]. In terms of its functional interpretation, the P600 has been viewed to reflect syntactic processing difficulty of different varieties, such as the inability of the parser to assign the preferred structure to the incoming words [22], syntactic reanalysis [29,30], or syntactic integration difficulty [31]. It has to be noted, however, that a purely syntactic interpretation of the P600 has been challenged by findings of P600 effects to semantic anomalies [32-36].

Other ERP-experiments reported a left anterior negativity ("LAN") being elicited by syntactic anomalies such as morphosyntactic violations [30,37,19,38] subcategorisation violations [38], and phrase structure violations [24,39]. Alternatively, LAN-effects have also been found for words that induce a larger memory load, either because of the lexical characteristics or (local) syntactic factors such as the triggering extra parsing steps [40,41].

To investigate whether number and gender mismatch between a pronoun and its antecedent is primarily a syntactic problem or a semantic problem, Osterhout and Mobley [42] registered ERPs during the processing of sentences in which the congruency of number or gender of a reflexive pronoun was manipulated ((1a) and (1b) respectively).

(1) a. The hungry guests helped themselves/himself* to the food.

b. The successful woman congratulated herself/himself* on the promotion.

Considering the syntactic rules (i.e. a reflexive pronoun has to be bound in its governing category and therefore they have to agree in number and gender with the antecedent), one can claim that the incongruent pronouns violate syntactic constraints. Alternatively, the incongruency can be regarded as a discrepancy of meaning of conceptual characteristics for number (i.e. *guests *being more than one person) as in (1a), and for biological gender characteristics (i.e. the female characteristics of a *woman*) as in (1b).

ERPs showed an enlarged widely distributed P600 in the absence of an N400 for both incongruent conditions (number and gender violation). Thus, the authors concluded that pronoun violations in number and gender are encountered as a syntactic or structure building problem rather than a semantic or meaning problem. In a second experiment in which personal pronouns were used (e.g., The aunt heard that *she/he *had won the lottery), gender mismatch also elicited an enlarged P600, but only when subjects judged such sentences to be unacceptable. N400 effects were found in sentences with semantic anomalous words (e.g. The boat sailed down the river and *barked*). The effects were replicated in a third experiment in which number agreement of reflexive pronouns and verbs were manipulated as well as semantic anomalous words were used. In a further study Osterhout, Bersick and McLaughlin [13] investigated the influence of stereotypical gender match between reflexive pronouns and the antecedent, as in *The surgeon prepared himself*_*match*_*/herself*_*mismatch*_*for the operation*. A surgeon is stereotypically male and reflexive pronouns that did not match with the probable gender of the antecedent elicited a late positivity similar to the P600 (but see [43] for conflicting evidence).

Our own group used German sentences in which the biological and/or syntactic gender of the pronoun was manipulated in combination with a non-diminutive or a diminutive antecedent [14]. In this situation, the type of antecedent influenced what kind of processes were involved. Whereas a biological gender violation between the pronoun and the non-diminutive antecedent resulted in an N400, no such effect was found for the sentences with a diminutive antecedent. Both, sentences with diminutive and non-diminutive antecedents with a syntactic gender violation caused a P600, which was more broadly distributed if the antecedent was a diminutive noun phrase and the pronoun was syntactically and biologically incongruent. The results indicate that for non-diminutives, both syntactic and conceptual information is used to establish coreference, while for diminutives the process might be more syntactically driven.

Most ERP studies focussed mainly on the coreferential relationship between the pronoun and the antecedent by manipulating the congruency in gender and/or number between the antecedent and the pronoun often resulting in either a violation or a less preferred disjoint coreferential relationship with a possible antecedent outside the available context [44,45]. There are however some studies that investigated pronoun resolution in relation to certain structural preferences in a wider discourse, e.g. by manipulating the distance between the pronoun and the antecedent [46], or structure parallelism [47]. It might therefore be argued that the use of violations in psycholinguistic ERP-paradigms is problematic, because the brain's handling of violations might well be different from the processing of correct items. To address the specific research question raised in the current paper, the violation approach is without alternative, however. In addition, it should be pointed out that during the natural use of language, violations can occur and may even be unavoidable. A case in point is our previous study of diminutives [14]. As diminutives denoting persons in German are assigned neuter gender (e.g. Das Männchen_neut _[the little man] from Der Mann_masc_), pronominal referral to such diminutive nouns entails either a syntactic (e.g. Das Männchen_neut _... er_masc_...) or a conceptual (e.g. Das Männchen_neut _...es_neut_...) violation.

Two ERP-reading studies, one in German and one in Dutch, are presented in this paper. In German and Dutch different overtly case-marked pronouns are used to refer to male and female persons. Furthermore, in a prepositional phrase, it is the preposition that assigns case to the pronoun. Thus, one can manipulate biological/syntactic gender (G) features separately from case-marking (C) features. In German, prepositions that assign either accusative or dative case were used, such that the accusative case (ihn_Masc_/sie_Fem_, in Eng.: him/her) was violated using dative case (ihm_Masc_/ihr_Fem_, in Eng.: him/her) and vice versa. In Dutch, however, prepositions assign object-case without differentiating between accusative and dative. Therefore, in the Dutch version of the experiment object-case (hem_Masc_/haar_Fem_, in Eng.: him/her) was violated using nominative case (hij_Masc._/zij _Fem._, in Eng.: he/she). Gender agreement was manipulated by presenting a female personal pronoun if the possible antecedent, i.e. the subject of the main clause, was male and vice versa. In Table 1 examples of sentences from all conditions are given in German and Dutch. By systematically combining pronouns with the same (congruent, G+) or different (incongruent, G-) gender as the antecedent with either correct (C+) or incorrect (C-) case assigned to the pronoun by the preposition in a prepositional phrase, four condition were created: correct case and gender (C+G+); incorrect case and congruent gender (C-G+); correct case and incongruent gender (C+G-); incorrect case and incongruent gender (C-G-).

Based on the results of the study of Coulson et al. [37] a LAN in combination with a P600 can be expected for the sentences with a case violation (C-G+, C-G-) in comparison to the correct sentence (C+G+), whereas based on our previous work an N400-P600 combination can be expected for the sentences with a gender mismatch (C+G-, C-G- compared to C+G+)[14].

## Results

### Results: German experiment

On average, subjects answered 92 % of the yes/no questions correctly (worst subject 86%) indicating no problems in understanding the sentences.

**Figure 1:**
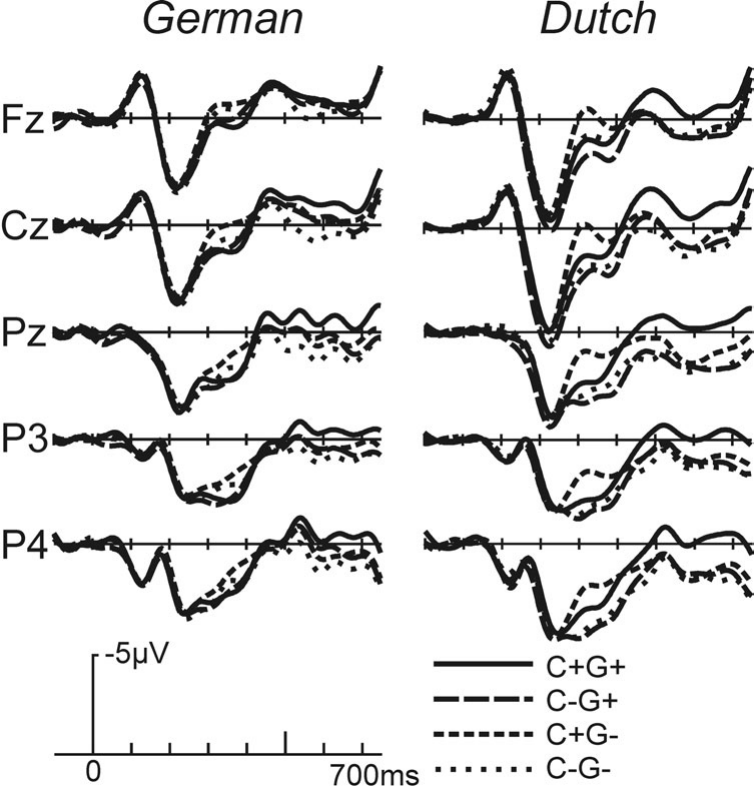
Grand average ERPs from both studies time locked to the onset of the pronoun at 5 electrode sites (baseline: 100 ms before the onset of the pronoun). Negative is plotted up in this and all subsequent figures. Conditions and their labels are illustrated in Table 1.

The grand average ERPs, time-locked to the onset of the critical pronoun are shown in Figure 1 (left column).

**Figure 2:**
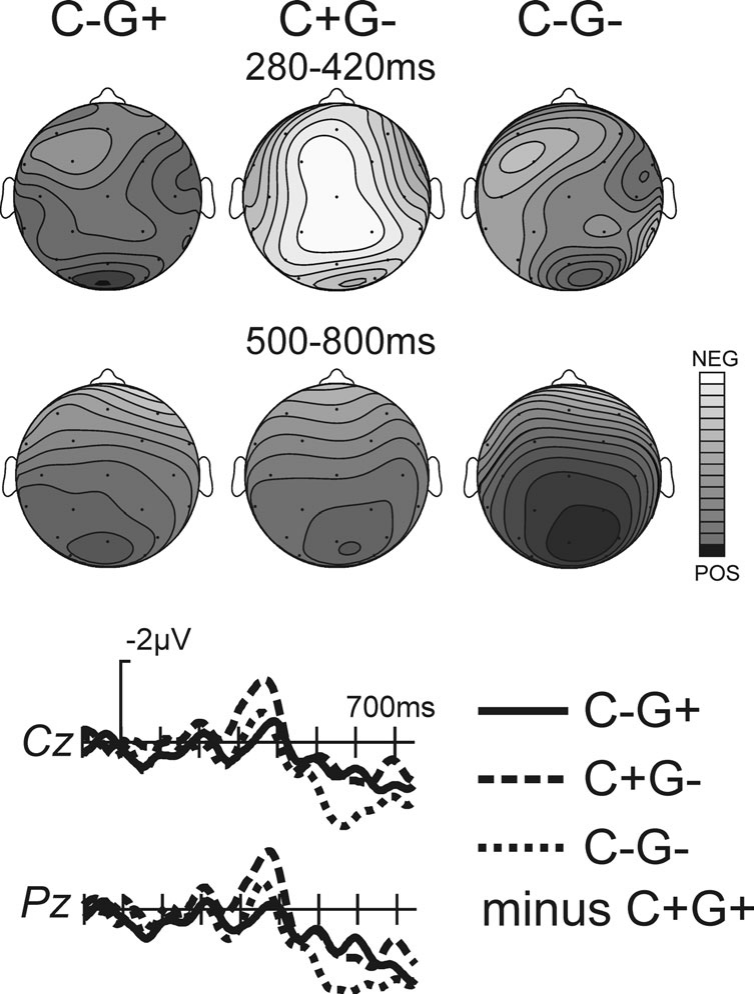
German study: Difference waves obtained by subtracting the correct condition waveforms from the various other conditions are illustrated at the bottom of the figure. The top row shows spline-interpolated isovoltage maps based on mean amplitude values of the difference waveforms (condition minus (C+G+)) in the 280–420 ms time window (these maps scaled with 0.3 μV as maximum and -1.1 μV as minimum). The middle row illustrates similar isovoltage maps for the 500–800 ms time-window, i.e. the P600 time range (these maps scaled with 1.6 μV as maximum and -0.7 μV as minimum).

Brain responses to conditions with either incorrect case or incongruent gender were different from those in the correct condition in two ways. For the condition with incongruent gender only (C+G-) a negativity can be noticed starting approximately at 280 ms after onset of the pronoun in comparison to the correct condition (C+G+). This deflection is broadly distributed and has a duration of approximately 150 ms (Figure 1). It is isolated by the computation of difference waves (Figure 2, bottom row) which form the basis of the topographic maps shown in Figure 2 (top row). The centroparietal maximum suggests that this effect is an instance of the N400. The (C-G-) condition shows a similar (but smaller) negativity. From 450 ms onwards ERPs from all incorrect conditions showed a positive shift relative to the C+G+ condition, which has a parietal maximum qualifying this effect as a P600 (Figure 2, medium row).

To quantify the negativity mean amplitudes in the 280 to 420 ms time-window were obtained. This window was defined by visual inspection of the effect in the grand average ERP signal and falls within the standard N400 time window (usually between 300–500 ms). An omnibus repeated measures ANOVA that crossed the factors 'Condition' (4levels: C+G+, C+G-, C-G+, C-G-) and 'Electrode sites' (29 sites) showed main effects of Condition (F(3,42) = 4.77; p(HF) ≤ .006) and Electrode sites (F(28,492) = 8.35; p(HF) ≤ .0001), but no significant interaction. Planned pair-wise comparisons computed for Cz, as the effect was largest at that site, showed that the amplitude of the negativity of the (C+G-)-condition was significantly larger in comparison to conditions with congruent gender (C+G+, C-G+) (see Table 2). There was no significant difference in the N400 time range between congruent gender conditions (C+G+ vs. C-G+). Although a negativity is apparent in the ERP-waveform for the incorrect case and incongruent gender condition (C-G-) in comparison to the congruent gender conditions (C+G+, C-G+), the corresponding comparisons did not reach significance (see Table 2).

For the P600, mean amplitude values were analysed for a time window of 500 to 800 ms. The respective omnibus ANOVA revealed main effects of Condition (F(3,42) = 3.13, p(HF) ≤ .0001) and Electrode sites (F(28,392) = 11.42, p(HF) ≤ .004) as well as an interaction between Electrode sites and Condition F(84,1176) = 3.91, p(HF) ≤ .0004).

To assess for amplitude differences of the P600, this global analysis was followed up by planned pair-wise comparison between conditions computed for data from the medial parietal site (Pz), where the P600 is maximal (see Figure 2 for difference waves [bottom row] and scalp distribution [medium row]) (Table 3).

### Discussion: German experiment

Two clear electrophysiological effects were present in the German data set: a relatively early negativity between 280 – and 420 ms, and a late positivity between 500 and 800 ms. The negativity was found for the gender incongruent/case correct (C+G-) condition, with a similar, smaller and statistically non-significant effect present for the double violation condition (C-G-). This negativity had a distribution similar to the "classical" N400 (cf. [48,49]), which is known to reflect problems of semantic integration. We take this as evidence that the processing of an incongruent gender pronoun causes problems of semantic/discourse integration. It is important to note, that in the current stimulus material biological/conceptual and syntactic gender of the antecedent coincided and were jointly violated. The processing system in this case seems to use the biological (conceptual/semantic) gender information in its attempt to establish a coreferential relationship: Because of the gender incongruency in the C+G- items it is not possible to build up a coreferential relationship between the pronoun and the only available possible antecedent in the discourse. The resulting integration problems cause an N400-effect.

Why, then, is there a smaller negativity for the C-G-condition? It seems that under these conditions the processes involved to resolve the incorrect case assignment (a syntactic problem) suppress attempts at lexical integration. The fact that the C-G- condition showed the largest P600 effect in the later time window raises the alternative possibility that the lack of an N400 effect might be due to an overlap with an enlarged positivity. We do not think that this is the case, because such an overlap effect would have led to differences in distribution of the P600 effect between the conditions. The P600 showed a similar distribution in the other two incongruent conditions (C+G- and C-G+), however. Finding a positivity for all three conditions indicates that both gender congruency and the violation of a local structural constraint disrupt pronoun resolution. We will return to the functional interpretation of this effect in the General Discussion.

### Results: Dutch experiment

**Figure 3:**
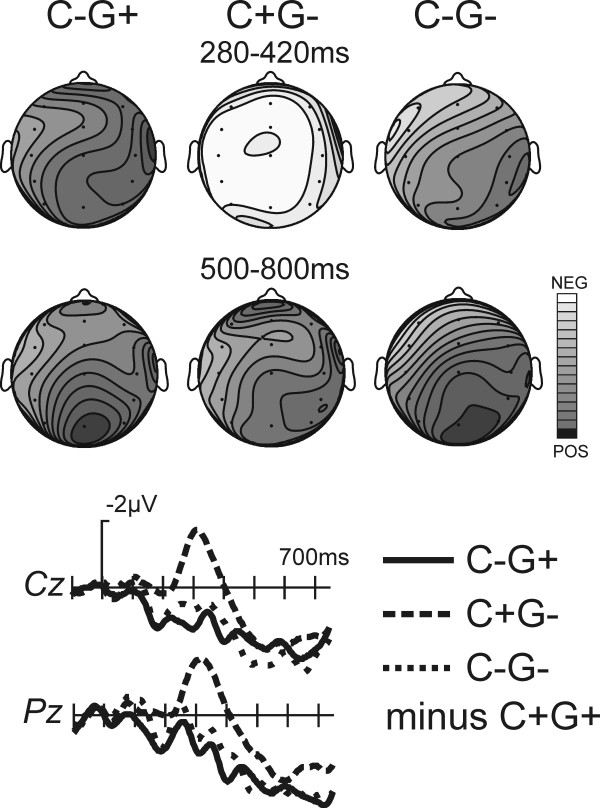
Dutch study: Difference waves obtained by subtracting the correct condition waveforms from the various other conditions are illustrated at the bottom of the figure. The top row shows spline-interpolated isovoltage maps based on mean amplitude values of the difference waveforms (condition minus (C+G+)) in the 280–420 ms time window (these maps scaled with 2.0 μV as maximum and -1.1 μV as minimum). The middle row illustrates similar isovoltage maps for the 500–800 ms time-window, i.e. the P600 time range (these maps scaled with 2.2 μV as maximum and -0.2 μV as minimum)

Subjects' answers to the yes/no questions were correct in 94 % (worst subject 88 %). Thus, subjects had no problems in understanding the sentences. Grand average ERPs are shown in Figure 1 (right column). As in the German study, the ERPs in the C+G- condition were associated with a negativity in the 280 to 420 ms range followed by a positive shift (see Figure 3 for difference waves [bottom row] and topographical maps for the negativity [top row] and positivity [medium row]).

The two conditions involving case incongruities (C-G+, C-G-) were associated with a positive shift as well. In this case, however, the ERPs to the incorrect conditions started to diverge from the ERPs in the correct condition as early as 280 ms.

To quantify these differences, mean amplitudes in the 280–420 ms time range were obtained. This window was defined based on prior ERP experiments investigating pronoun resolution that had revealed a negativity in this time range [14,45]. The time-window was corroborated by visual inspection as well as by calculating the onset and ending of the divergence using pair wise comparisons between the correct condition and the correct case incongruent gender condition (C+G+ vs C+G-) in consecutive windows of 12 ms each (3 data points) on Fz, Cz and Pz separately. The first window started at the onset of the pronoun; the next window moved 4 ms (1data point) and therefore overlapping 8 ms (2 data points) with the previous window. To minimize the danger of false positives, the 12 ms windows were only considered significant when three successive windows showed these effects (p < .05), resulting in the above-mentioned time frame of 280–420 ms after pronoun onset.

This window overlaps with the window in which the negativity was found in the German study. The omnibus repeated measures ANOVA for this 280–420 time range showed a main effect of Condition (F(3, 45 = 5.92; p(HF) ≤ .0061), as well as an interaction of Electrode sites × Condition (F(84,1260 = 2.32; p(HF) ≤ .0188). Planned pair-wise comparisons at the Cz electrode, near the maximum of the negativity, revealed that the negativity seen in the C+G- condition was significant in comparison to the other three conditions. As can be seen in Figure 1 (right column) and Figure 3, the C-G- and C-G+ conditions showed an early positivity relative to the correct condition (C+G+) upon visual inspection, which was partially confirmed by the pair-wise comparisons (see Table 4).

As in the German study, the negativity found for the correct case incongruent gender condition (C+G-) was followed by a positive shift. The isovoltage maps show that the positivity in the conditions in which case is violated (C-G+, C-G-) has a centroparietal maximum, thus resembling a P600 (Figure 3, medium row). To evaluate this effect mean amplitudes (time window 500–800 ms) were entered into an omnibus ANOVA, which showed main effects of Condition (F(3,45) = 5.92, p(HF) ≤ .002) and Electrode sites (F(28,420) = 8.30, p(HF) ≤ .001) as well as an Condition × Electrode sites interaction (F(84,1260) = 2.32, p(HF) ≤ .02). This was followed up by pair-wise comparisons at Pz, where the effect was maximal (Table 5). A significant difference was found between the correct condition (C+G+) and each of the other conditions.

### Discussion: Dutch experiment

As in the German study, the negativity of the C+G-condition clearly resembles an N400 thus indicating problems with the integration of semantic/discourse information. Finding a negativity for the condition in which gender only was violated and a positive shift for the conditions with a case violation clearly indicates different underlying processes in pronoun comprehension of either a mismatch in gender between the pronoun and the antecedent and/or incorrect case marking.

The particular case violation used in the Dutch study is obviously detected as early as 280 ms after the information becomes available. This is the same moment at which the semantic/discourse integration problems in the C+G- condition are apparent. Similar early positivities have been reported in several studies in which the processing of less preferred structures over highly preferred structures was investigated (cf. [27,50,25]. For example, in a Dutch reading study of Lamers [50,51] in which subject/object ambiguities were disambiguated by a case marked pronoun a similar positivity occurred at a nominative case marked pronoun as the second NP, disambiguating the sentence in a less preferred object initial sentence, as in *The old woman in the street took-care of him/he*. This indicates that nominative object case violation on a personal pronoun, which is a closed class word, influences comprehension processes as early as 280 ms.

In the later time frame all three conditions with a gender incongruency and/or a case violation in comparison to the correct condition (C+G+) showed a late positive shift broadly distributed over the scalp. As in the German study, this indicates that both gender congruency and correctness in case marking affects pronoun resolution in this later time frame probably involving the final structure building of the sentence.

In summary, the Dutch study showed that gender information not only affect semantic/discourse integration processes, but also effect later processes that are merely syntactic in nature. Case information influences the pronoun resolution as early as the lexical/discourse integration problems elicited by the gender incongruency become apparent. Additionally gender incongruency as well as the violation of the case assignment by the preposition influences later processes possibly related to the final structure building process.

## Discussion

The electrophysiological effects found in the German and the Dutch study provide evidence that in both languages semantic and syntactic gender information is used during pronoun resolution, and that the pronoun resolution is influenced by the case assignment under preposition governing. As expected, in both studies an N400-P600 complex was observed, corroborating earlier findings of Schmitt et al. [14] that both, syntactic and semantic integration processes play a role during pronoun processing. The N400 was found for the condition with a gender incongruency only (C+G-), indicating a problem of semantic/discourse integration whenever there is a gender mismatch between the pronoun and the only possible available antecedent which makes it impossible to build up a coreferential relationship. In addition, a P600 was found in both studies in all incongruent conditions compared to the C+G+ condition. With regard to the P600 in the C+G-, it is clear that this positivity is the final consequence of the gender mismatch. It is not possible to conclude whether the P600 is a reflection of the coindexing problem or rather driven by problems to build the most preferred final structure, namely a structure in which a coreferential relationship between the pronoun and the antecedent is established. An interpretation of the processes underlying the P600, which seems to fit the current set of data quite well, has been given recently by Hagoort in the context of his Memory, Unification, and Control (MUC)-model of language [52]. In his interpretation, Hagoort is drawing heavily from the 'Unification model', a computational model formulated by Vosse and Kempen [53]. The key concept of this model is that each word in the lexicon is associated with a structural frame that specifies the possible structural environment of this particular word. In the process of sentence comprehension, the syntactic frames are unified into a structural representation of the whole sentence by the formation of 'unification links'. Specifically, Hagoort proposes that the P600 latency should be related to the time needed to establish unification links of sufficient strength. "The time it takes to build up the unification links until the required strength is reached, is affected by ongoing competition between alternative unification options (syntactic ambiguity), by syntactic complexity, and by semantic influences." The amplitude of the P600 on the other hand is thought to vary as a function of the amount of competition, which should be reduced when the number of alternative unification options is smaller, or when lexical, semantic or discourse information biases unification in a particular direction. In this framework, the greater P600 amplitude in the C-G- condition (compared to C-G+) in the German study could be attributed to the fact that in this condition the strength of the unification process is not only affected by the gender incongruency, but also by the case violation, causing unsuccessful unification attempts.

Although the gender manipulation in the German and Dutch study was identical, a different case manipulation was used due to the differences in the case marking system of personal pronouns of the two languages. It turns out that the one remarkable difference between the two current experiments mainly concerns those conditions in which case assignment was violated. In the German study accusative case was violated by wrongly assigning dative case and vice versa, whereas in the Dutch study object case was substituted by nominative case (Although it is practically possible in German to wrongly assign nominative case instead of the correct accusative or dative case, it was not considered as an alternative, because it would have been necessary to use only prepositions that assign or accusative case or dative case, but not both prepositions. In addition, the feminine nominative and accusative personal pronoun are homophones, and also ambiguous with the third person plural nominative and accusative personal pronoun, i.e. in all cases "sie"). In the Dutch study, the positivities found for the conditions in which incorrect case was assigned (C-G+, C-G-) started as early as 280 ms after the onset of the pronoun, whereas in the German study the onset latency was approximately 500 ms. It thus appears that the nominative case violation is more salient than the accusative/dative case violation, because the nominative case in Dutch (as well as in German) is not morphologically marked, whereas object case in Dutch and accusative and dative case in German are. This probably makes the nominative violation easier recognizable, and thus, affects the onset of the involved processes. In addition, nominative case cannot be assigned under governing of a preposition, whereas dative and accusative case both are. Thus in case of a nominative pronoun, it clearly is the incorrect (non)marking of the pronoun that causes the violation. In German however, although the incorrect marking becomes available at the pronoun, it can also be that the proceeding preposition is the actual item that does not fit, especially since there are prepositions that do assign the case of the actually form of the pronoun.

Both the saliency of the nominative case in a prepositional phrase in comparison to the case marked accusative/dative case marked pronoun in German, and the possible dual cause of the violation in the German study (i.e. wrong preposition and/or wrong case marking) influence the number of alternative unification options and the timing of these operations. This, then, might explain the latency difference of the positivities in the two studies.

It should be pointed out that Hagoort's MUC framework delivers just one of several competing accounts of the processes underlying the P600. It provides, however, a useful explanation as to why the double violation condition (C-G-) gives rise to a greater P600. A monitoring perspective as taken by van Herten et al. [34] might handle our set of data equally well. These authors propose that after encountering an unexpected linguistic event, the reader reattends the unexpected unit to check upon its veridicality. A linguistic event can be unexpected for several reasons and the degree of expectedness is reflected in the characteristics of the P600.

The differences in amplitude and onset of the P600 found in the two current studies are harder to accommodate by accounts envisioning the P600 as a reflex of purely syntactic integration difficulty [31] or syntactic reanalysis [29].

Although for both studies not only an N400 effect and a P600 was predicted, but also a LAN, no evidence was found for such an effect. In comparison to English, the language used in most other ERP studies investigating pronoun resolution, Dutch and German have a relatively free word order, whereas English has not [13,37,43]. As argued before, language specific characteristics can cause differences in parsing and comprehension processes. What is more, in the studies reported in this paper, there was one and only one possible antecedent, and the pronoun received case from the preposition in the prepositional phrase. By contrast, Coulson et al. [37] not only used pronouns that do not need an antecedent that is actually mentioned in the discourse (e.g. first person plural), they also manipulated possessive pronouns following a transitive verb that could not take a person as a second argument, as was illustrated in example 2. Given the presence of a possible antecedent and a third person pronoun in the present study, the differences between the effects found in the current study and the study of Coulson et al. might be explained by the differences in pronoun resolution. Whereas in the current study it is very likely that an attempt is made to establish a coreferential relation ship between the pronoun and the antecedent given in the preceding context, this is not the case for a first or second person pronoun as used by Coulson et al.

To summarize, in German and Dutch not only gender information affects the resolution of a pronoun, but also local structural constraints (i.e. case-assignment by a preposition) influence the parsing process. The differences in the characteristics of the effects indicate that case information influences processes merely syntactic in nature, whereas gender information also affects semantic/discourse integration processes. Evidence was found that a combination of incorrect case assignment with a gender incongruency affect the resolution processes as early as 280 ms, and involves more processing activity than a pronoun of which only one source of information is violated or incongruent. Additionally it was argued that the features of the case violation in combination with language specific characteristics play an important role in the onset and nature of the processes involved in pronoun resolution.

## Conclusion

To the extent that the assignment of semantic and syntactic processes to ERP negativities (i.e., the N400) and positivities (i.e., the P600) is valid, the presence of both effects in conditions with a gender violation indicates that pronoun resolution with gender incongruency between the pronoun and the antecedent results in semantic as well as syntactic integration problems. Violations in the sense of incorrect case marking without a gender violation give rise to syntactic but not semantic integration problems as attested by the presence of a positivity. Both case and gender information appear to be used as they come available.

## Methods

### Subjects

Fifteen native speakers of German (age range: 19–27 years; 12 women) and 16 native speakers of Dutch (age range: 19–24 years; 14 women) were recruited from the student populations at the Universities of Magdeburg, Germany, and Maastricht, The Netherlands. All were neurologically healthy, right handed and paid for their participation in a single session. Too many artefacts necessitated the rejection of one additional German subject.

### Material

The same set up of materials, procedure, and analyses were followed for the German and Dutch versions of the experiment unless mentioned otherwise. A total of 128 nouns specifying persons' professions, titles or states were selected. Half of these nouns represented clearly male, the other half clearly female persons. These nouns served as the subject in sentences consisting of a main clause and a subordinate clause. In the subordinate clause the complementizer was followed by an auxiliary or link verb, which was followed by a general subject (*man*, *jemand *in German; *men*, *iemand *in Dutch; which can be translated as *someone *in English), which was followed by a prepositional phrase including a pronoun that referred to the subject of the main clause. As described above, the case of the pronoun was congruent or incongruent (C+ or C-) and the gender matched or mismatched with the gender of the antecedent (G+ or G-). This resulted in four conditions (Table 1). The pronoun was followed by an infinitive verb. In the German version half of the prepositions assigned accusative case, the other half dative case equally divided over sentences with a male or female NP as the subject of the main clause. In Dutch all prepositions assigned general object case.

### Procedure

Four blocks of 32 experimental sentences (i.e. 8 sentences of each condition) and 30 filler sentences were visually presented in a word-by-word fashion (350 ms word presentation, 300 ms blank screen) in the middle of a video-screen. To check whether the subjects were actually reading the sentences 10 experimental sentences and 10 filler sentences occurring at random positions within a block were followed by a simple yes/no question addressing the content of the first part of the sentence, which had to be answered by a button-press. To avoid interference with the resolution of the pronoun, care was taken that the question never addressed the pronoun directly nor the prepositional phrase. If the question was related to the subject of the main clause, the same NP was used in the question to prevent a confound of biological gender (e.g. The girl had the flu, and therefore Question: Was the girl sick? y/n). Words were presented in 16 points font size. After the last word of a sentence a blank screen was shown for 600 ms, followed by a fixation asterisk (1750 ms on the screen, 300 ms blank screen). Each block lasted approximately 15 minutes. The entire experiment, including electrode application and removal took 2.5–3 hours.

Subjects were tested individually in a dimly lit sound attenuating room facing a colour video screen at a distance of 110 cm. They were instructed to move as little as possible and to read the sentences for content. They were allowed to blink between sentences as soon as an asterisk appeared on the screen and while answering a question. After each block the subjects received feedback on their performance on the questions.

### Data acquisition and analysis

In the German study continuous EEG was recorded from 29 scalp sites including all standard sites of the international 10/20 system using tin electrodes in an electro-cap. The electrode sites were: Fp1/2, F3/4, C3/4, P3/4, O1/2, F7/8, T7/8, P7/8, Fz, Cz, Pz, Fc1/2, Cp1/2, Po3/Po4, Fc5/6, Cp5/6. In the Dutch study continuous EEG was recorded at 29 scalp sites: Fp1/2, F3/4, C3/4, P3/4, O1/2, F7/8, FT7/8, TP7/8, T7/8, P7/8, Fz, Cz, Pz, Oz, Fcz, Fc3/4, Cp3/4. In both studies bio-signals were recorded with a left mastoid reference, and were re-referenced off-line to the mean of the activity at the two mastoid processes. Bipolar EOG was recorded between electrodes at the outer left and right canthus and above the eyebrow and below the left eye. The impedance of the electrodes was kept under 5 kOhm. The signals of each electrode were amplified (time constant 10 s), bandpass filtered between .05 and 30 Hz, and digitised with a sampling frequency of 250 Hz. The signal was monitored for artefacts, such as eye-movements. The baseline of the waveforms was adjusted on the basis of the averaged activity 100 ms preceding the pronoun onset. From the continuous signal epochs were created of 1024 ms starting 100 ms prior to pronoun onset. Due to eye movements, blinks, and electrode drift approximately 7 % of the trials were rejected in the German experiment, whereas this was the case in 25 % in the Dutch experiment with no difference across conditions in both studies. Averages were computed across all remaining trials per condition. Subsequent repeated measures ANOVAs used mean amplitude values (relative to the 100 ms pre-stimulus baseline) computed for each subject and each condition. The Huynh-Feldt epsilon correction was used, when evaluating effects with more than one degree of freedom in the numerator to adjust for sphericity violations. The original degrees of freedom and the corrected p-values are reported.

## Abbreviations

C+G+: case congruent, gender congruent

C-G+: case incongruent, gender congruent

C+G-: case congruent, gender incongruent

C-G-: case incongruent, gender incongruent

ERP: event-related potential

MUC: memory, unification and control

NP: noun phrase

## Authors' contributions

MJAL co-designed the study, performed and analysed the experiments, and wrote the first draft of the manuscript, BMJ co-designed the study, provided psycholinguistic input and co-wrote the first draft, AH performed part of the experiments and performed some statistical analyses, TFM co-designed the study and wrote the final draft of the manuscript. All authors read and approved the final manuscript.

## Figures and Tables

**Figure 4:**
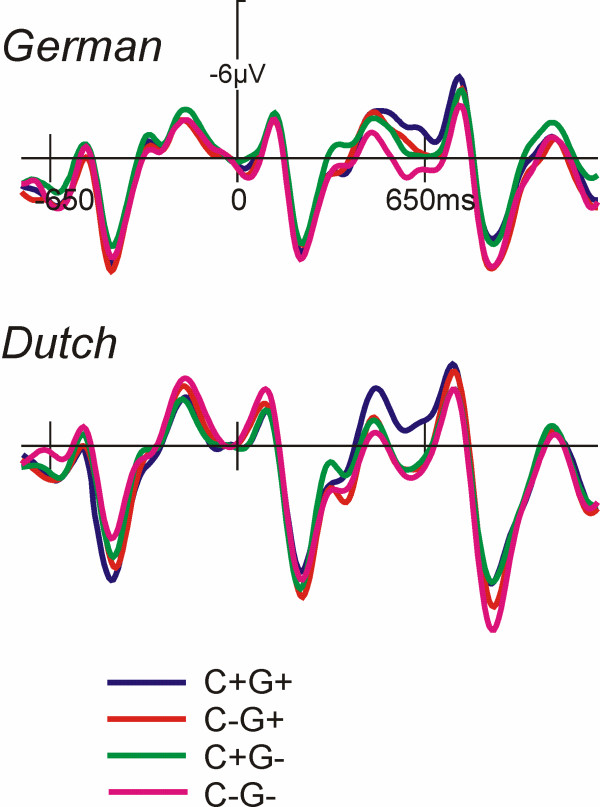
Group average potentials from both studies (Cz-site) plotted with a longer time-base. The 100 ms prior to the appearance of the pronoun (time = 0) were used for a baseline. The tick-marks mark the onset of the words. There are no systematic effects prior to the pronoun in both experiments. Also, no effects are observed for the word following the pronoun.

**Table 1:** Example materials for the German (upper part) and the Dutch experiment (lower part).

**Condition**	**German sentences**
1. C+G+ 2. C-G+ 3. C+G- 4. C-G-	**Der Kumpel**_**male **_war eingeladen und darum hat man mit **ihm**_**Masc-Dat **_gerechnet. *Der Kumpel_**male **_war eingeladen und darum hat man mit ***ihn***_***Masc-Acc ***_gerechnet. **Der Kumpel**_**male **_war eingeladen und darum hat man mit ***ihr***_***Fem-Dat ***_gerechnet. *Der Kumpel_**male **_war eingeladen und darum hat man mit ***sie***_***Fem-Acc ***_gerechnet.
**Translation:**	the friend_male _was invited and therefore have they on him_Dat_/*him_*Acc*_/*her*_*Dat*_/*her_*Acc *_counted "The friend was invited and therefore they did count on him/*him/*her*/*her being present."

**Condition**	**Dutch sentences**

1. C+G+ 2. C-G+ 3. C+G- 4. C-G-	**De vriend**_**male **_was uitgenodigd en daarom heeft men op **hem**_**Masc-Obj **_gerekend. *De vriend_**male **_was uitgenodigd en daarom heeft men op ***hij***_***Masc-Nom ***_gerekend. **De vriend**_**male **_was uitgenodigd en daarom heeft men op ***haar***_***Fem-Obj ***_gerekend. *De vriend_**male **_was uitgenodigd en daarom heeft men op ***zij***_***Fem-Nom ***_gerekend.
**Translation:**	the friend_male _was invited and therefore have they on him_*Obj*_/*he_*Nom*_/*her*_*Obj*_/*s*he*_*Nom *_counted "The friend was invited and therefore they did count on him/*he/*her*/*s*he *being present."

Dat = Dative Nom = Nominative Acc = Accusative Obj = Object-case Fem = Feminine Masc = Masculine C = Case G = Gender + = correct/congruent - = incorrect/incongruent

**Table 2:** Overview of the p-values of the planned pair-wise comparisons between the four conditions of the German study of the data in the 280–420 ms window at Cz; n.s. = not significant, p > .05.

**Cz **	**C-G+**	**C+G-**	**C-G-**
**280–420 ms**			
**C+G+**	n.s.	**.006**	n.s
**C-G+**		**.0003**	n.s.
**C+G-**			**.015**

**Table 3:** Overview of the p-values of the planned pair-wise comparisons between the four conditions of the German study of the data in the 500–800 ms time-window at Pz; n.s. = not significant, p > .05.

**Pz **	**C-G+**	**C+G-**	**C-G-**
**500–800 ms**			
**C+G+**	**.02**	**.02**	**.001**
**C-G+**		n.s.	**.04**
**C+G-**			**.02**

**Table 4:** Overview of the p-values of the planned pair-wise comparisons between the four conditions of the Dutch study of the data in the 280–420 ms window at Cz; n.s. = not significant, p > .05.

**Cz **	**C-G+**	**C+G-**	**C-G-**
**280–420 ms**			
**C+G+**	**.001**	n.s.	n.s
**C-G+**		**.0004**	n.s.
**C+G-**			**.0042**

**Table 5:** Overview of the p-values of the planned pair-wise comparisons between the four conditions of the Dutch study of the data in the 500–800 ms time-window at Pz; n.s. = not significant, p > .05.

**Pz **	**C-G+**	**C+G-**	**C-G-**
**280–420**			
**C+G+**	**.0001**	**.003**	.0001
**C-G+**		n.s.	n.s.
**C+G-**			n.s
